# Deep learning prediction of BRAF-RAS gene expression signature identifies noninvasive follicular thyroid neoplasms with papillary-like nuclear features

**DOI:** 10.1038/s41379-020-00724-3

**Published:** 2020-12-10

**Authors:** James M. Dolezal, Anna Trzcinska, Chih-Yi Liao, Sara Kochanny, Elizabeth Blair, Nishant Agrawal, Xavier M. Keutgen, Peter Angelos, Nicole A. Cipriani, Alexander T. Pearson

**Affiliations:** 1 Department of Medicine, Section of Hematology/Oncology, University of Chicago Medical Center, Chicago, IL USA; 2 Department of Pathology, University of Chicago Medical Center, Chicago, IL USA; 3 Department of Surgery, University of Chicago Medical Center, Chicago, IL USA

**Keywords:** Pathology, Diagnostic markers, Head and neck cancer, Thyroid diseases

## Abstract

Noninvasive follicular thyroid neoplasms with papillary-like nuclear features (NIFTP) are follicular-patterned thyroid neoplasms defined by nuclear atypia and indolent behavior. They harbor *RAS* mutations, rather than *BRAF*^V600E^ mutations as is observed in papillary thyroid carcinomas with extensive follicular growth. Reliably identifying NIFTPs aids in safe therapy de-escalation, but has proven to be challenging due to interobserver variability and morphologic heterogeneity. The genomic scoring system BRS (BRAF-RAS score) was developed to quantify the extent to which a tumor’s expression profile resembles a *BRAF*^V600E^ or *RAS*-mutant neoplasm. We proposed that deep learning prediction of BRS could differentiate NIFTP from other follicular-patterned neoplasms. A deep learning model was trained on slides from a dataset of 115 thyroid neoplasms to predict tumor subtype (NIFTP, PTC-EFG, or classic PTC), and was used to generate predictions for 497 thyroid neoplasms within The Cancer Genome Atlas (TCGA). Within follicular-patterned neoplasms, tumors with positive BRS (RAS-like) were 8.5 times as likely to carry an NIFTP prediction than tumors with negative BRS (89.7% vs 10.5%, *P* < 0.0001). To test the hypothesis that BRS may serve as a surrogate for biological processes that determine tumor subtype, a separate model was trained on TCGA slides to predict BRS as a linear outcome. This model performed well in cross-validation on the training set (*R*^2^ = 0.67, dichotomized AUC = 0.94). In our internal cohort, NIFTPs were near universally predicted to have RAS-like BRS; as a sole discriminator of NIFTP status, predicted BRS performed with an AUC of 0.99 globally and 0.97 when restricted to follicular-patterned neoplasms. *BRAF*^V600E^-mutant PTC-EFG had BRAF^V600E^-like predicted BRS (mean −0.49), nonmutant PTC-EFG had more intermediate predicted BRS (mean −0.17), and NIFTP had RAS-like BRS (mean 0.35; *P* < 0.0001). In summary, histologic features associated with the BRAF-RAS gene expression spectrum are detectable by deep learning and can aid in distinguishing indolent NIFTP from PTCs.

## Introduction

Thyroid neoplasms can be broadly categorized as papillary carcinomas or follicular-patterned neoplasms. Classic papillary thyroid carcinoma (PTC-classic) are infiltrative, often metastasize to lymph nodes, and frequently harbor *BRAF*^V600E^ mutations (~45%), *RET*-*PTC* rearrangements (~20%), and/or a *BRAF*^V600E^-like gene expression signature [1–3]. Follicular thyroid neoplasms have been described with several historical frameworks. Due to high interobserver variability and observed behavioral heterogeneity, definitions of the various follicular-patterned neoplasms have come under recent attention. Modern descriptions of follicular-patterned neoplasms (excluding conventional follicular adenomas and follicular carcinomas) include three main types: PTC with extensive follicular growth (PTC-EFG), noninvasive follicular thyroid neoplasms with papillary-like nuclear features (NIFTP), and invasive encapsulated follicular variant of PTC (IE-PTC-FV) [4, 5].

1. PTC-EFG neoplasms (alternate nomenclature includes the infiltrative follicular variant of PTC, or PTC with prominent follicular architecture) are characterized by predominantly follicular architecture, infiltrative growth similar to classic PTC, and lymphatic metastases. They may have focal papillae and usually demonstrate intranuclear pseudoinclusions. Like classic PTCs, they have been observed as having a high frequency of *BRAF*^V600E^ (but not *RAS*) mutations.
2. NIFTP is a relatively new classification (2016) with recently updated diagnostic criteria (2018). They are defined by follicular growth, circumscription, and indolent behavior. They possess nuclear features similar to PTCs. Unlike PTCs, however, these neoplasms contain frequent *RAS* (but not *BRAF*^V600E^) mutations [2, 5–15].
3. IE-PTC-FV are histologically and genetically similar to NIFTP, with follicular growth, papillary-like nuclei, and *RAS* mutations, but demonstrate either capsular, vascular, or intrathyroidal invasion which precludes the diagnosis of NIFTP. IE-PTC-FV are associated with more aggressive behavior, with a capacity for hematogenous metastases similar to follicular carcinoma.

Identification of NIFTP is clinically relevant, as their indolent course allows for de-escalation of therapy compared to conventional PTC. As a result of their indolent behavior, NIFTPs are less aggressively managed, and neither thyroidectomy nor radioactive iodine ablation are recommended [16]. However, diagnosis of NIFTP can be challenging due to the variability of nuclear features, geographic heterogeneity within each tumor, and propensity for inter-observer variability due to differing diagnostic thresholds among pathologists [17–19].

The most recently proposed diagnostic criteria for NIFTP include: (1) follicular growth with no papillae or psammoma bodies and <30% solid/trabecular growth, (2) encapsulation or clear demarcation with no vascular or capsular invasion, (3) absence of tumor necrosis and mitoses <3 per 10 high-power fields, and (4) significant nuclear features [14, 15]. Nuclear features are defined as demonstration of sufficient abnormality in at least two of three categories: (1) size and shape (enlarged or elongated), (2) membrane irregularities (grooves, folds, pseudoinclusions), or (3) chromatin irregularities (glassy nuclei, chromatin clearing or margination) [4]. In an additional effort to correlate behavior to genetics, presence of *BRAF*^V600E^ mutation, BRAF VE1 immunopositivity, or high-risk mutation (*TERT* promoter, *TP53*) excludes a diagnosis of NIFTP [15].

While the diagnostic criteria for NIFTP have helped standardize the approach towards recognizing and diagnosing this indolent entity, the problem of interobserver variability is not entirely ameliorated. While NIFTP neoplasms are overall exceptionally indolent, some neoplasms diagnosed as NIFTP have been rarely observed to metastasize, highlighting the challenge of excluding rare aggressive neoplasms that may otherwise be classified as NIFTP under these diagnostic guidelines [20]. Furthermore, these criteria have not been well-established in the setting of oncocytic morphology, multifocality, or in subcentimeter lesions. However, some authors suggest that large (>4 cm), small (<1 cm), and oncocytic neoplasms otherwise meeting criteria for NIFTP should be considered part of the NIFTP spectrum [21–23]. Finally, there has been recognition that a more “biologically accurate” definition of the indolent NIFTP variant should be pursued, as the current definition fails to recognize the variety of biologic behavior in thyroid neoplasms [4]. While the presence of *RAS* mutations in NIFTPs and *BRAF*^V600E^ mutations or *RET*-*PTC* fusions in PTCs appear specific, the prevalence of these findings is not high enough to consistently guide diagnosis.

In a broad genomic characterization of thyroid cancers in The Cancer Genome Atlas (TCGA),  Cancer Genome Atlas Research Network developed BRAF-RAS score (BRS), a 71-gene expression signature designed to quantify the extent to which the gene expression profile of a given tumor resembles either the *BRAF*^V600E^ or *RAS*-mutant profiles [1]. The score is scaled from −1 to +1, with negative scores indicating BRAF^V600E^-like signature and positive scores indicating RAS-like expression. The development of BRS has introduced the ability to assess whether the gene expression of a non-mutant thyroid neoplasm is similar to that of a *BRAF*^V600E^ or *RAS* mutant, highlighting the fact that tumors could possess a variety of genomic or epigenetic alterations resulting in activation of the same oncogenic pathways driven by *BRAF*^V600E^ or *RAS* mutations. In addition to being highly specific for *BRAF*^V600E^ or *RAS* mutations, BRAF^V600E^-like gene expression (negative BRS) identified *BRAF* fusions, *RET* fusions, and was enriched for tumors with poor differentiation, classical and tall cell histology, and ERK pathway activation. RAS-like expression (positive BRS) was seen mostly in follicular tumors and corresponded to predicted PI3K/AKT and MAPK signaling.

Deep learning tools are becoming increasingly popular for histologic analyses and have been used to predict tumor biomarker status, clinical variables, tumor subtypes, and mutation status in a variety of cancers [24–27]. In thyroid cancer specifically, several groups have demonstrated that histologic features associated with *BRAF*^V600E^ and *RAS* mutations are detectable using deep learning [28, 29]. However, it is not yet known whether non-mutant BRAF^V600E^-like or RAS-like tumors, as defined by the BRS, retain similar histologic features to their mutated counterparts, nor is it known whether these image features have any relevance to the diagnosis of follicular-patterned thyroid neoplasms.

In light of the observation that NIFTP are associated with *RAS* mutations while PTC-EFGs tend to have *BRAF*^V600E^ mutations, we hypothesized that NIFTPs possess RAS-like gene expression signatures, even in the absence of a *RAS* mutation, which portend phenotypic similarity to their mutated counterparts. If true, this could allow for the use of BRS to help distinguish between NIFTPs and PTCs with extensive follicular growth. We thus sought to investigate whether consideration of BRAF-RAS spectrum gene expression could aid in the distinction between subtypes of follicular-patterned neoplasms.

## Materials and methods

### Data preparation

**Fig. 1:**
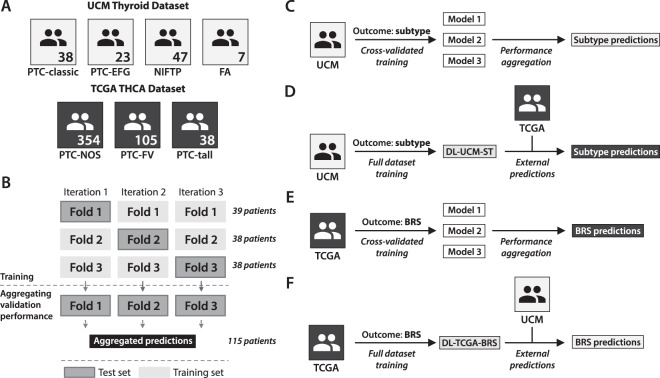
Deep learning experimental strategy. **a** Distribution of thyroid neoplasm subtypes within the institutional (University of Chicago Medicine, UCM) and TCGA datasets. The institutional dataset contained a total of 115 slides across four subtypes, as detailed here. The TCGA thyroid cohort (THCA) contained 497 slides across three subtypes: papillary thyroid carcinoma, not otherwise specified (PTC-NOS), papillary thyroid carcinoma, follicular variant (PTC-FV), and papillary thyroid carcinoma, columnar cell variant (PTC-tall). As the annotated subtypes in TCGA use an older naming convention before the recognition of NIFTP, these annotations cannot be directly compared between datasets. **b** Cross-validation plan for training a deep learning model. A deep learning model was first trained on the institutional dataset to predict tumor subtype, using threefold cross-validation as illustrated here. Patients were separated into three categories of 39, 38, and 38 patients per category, with annotated subtypes balanced between categories. For each of three iterations, two-thirds of the dataset was used for training and one-third was used for validation to generate predictions. Predictions in each of the left-out validation sets were aggregated across the three *k*-fold iterations in order to generate the reported cross-validation performance statistics. **c** Overview of the deep learning training strategy used to generate cross-validated subtype predictions on the institutional dataset, as also described in (**b**). **d** After cross-validation was completed, a final model (DL-UCM-ST) was trained across the entire institutional dataset to predict subtype. This model was applied to the TCGA dataset, for which subtype predictions were generated. **e** Deep learning models were trained on the TCGA dataset to predict BRS, using cross-validation as described in (**b**). **f** After cross-validation was completed, a final model (DL-TCGA-BRS) was trained across all TCGA slides to predict BRS. This model was then used to generate BRS predictions on the institutional dataset.

H&E-stained slides were prepared for 115 neoplasms, with a distribution of diagnoses listed in Table 1 and Fig. 1a, were scanned at 40x (using either Philips Ultra Fast Scanner 1.8 (Philips, Best, The Netherlands) or Aperio ScanScope XT (Leica Biosystems, Buffalo Grove, IL, USA)), and were digitally annotated by pathologists (AT, NAC) with regions of interest (ROI) encircling tumors. Image tiles of size 299 pixels x 299 pixels were extracted from slides in a grid-wise pattern within corresponding ROI at 302 μm × 302 μm (effective optical magnification: ×10). Background tiles were filtered by examining each image tile in the HSV color space, identifying “grayspace” pixels by counting the number of pixels with a hue value less than 0.05, and discarding the tile if the grayspace fraction was above 50%. Digital tile images were then standardized with Tensorflow to give each image a mean of zero and variance of one [30].

**Table 1:** Distribution of thyroid cancer subtypes in institutional dataset.

Subtype	Abbreviation	# of slides
Papillary thyroid carcinoma, classic	PTC-classic	38
Papillary thyroid carcinoma, extensive follicular growth	PTC-EFG	23
Noninvasive follicular thyroid neoplasm with papillary-like nuclear features	NIFTP	47
Benign follicular adenomas	FA	7

### Training deep learning models on an institutional dataset

Deep learning models were trained on extracted image tiles from our institutional dataset using an Xception-based network in Tensorflow, trained to predict tumor subtype [31]. Category-level balancing was used when generating batches for training, such that each batch contained equal proportion of tiles from each category in an attempt to reduce bias. Before training, data were separated into three equal *k-*folds, and a brief hyperparameter search was performed by training with 2/3 of the data and validating on the left out one-third. Hyperparameters evaluated included all possible combinations of learning rate (0.0001, 0.001), batch size [8, 16], post-convolution pooling (“max” vs “average”), number of post-convolution hidden layers (0, 1) and epochs [1–10]. The hyperparameter search did not include further parameters or a wider breadth of parameter searching due to computational constraints. After this brief search, hyperparameters were selected as detailed in Supplementary Table S1, and threefold cross validation was finished with these parameters (Fig. 1b). Predictions were generated on held-out validation sets for each *k*-fold. AUCs for each category were generated by varying the number of predicted-positive tiles needed to result in a positive slide-level prediction. A final model was then trained to the entire dataset (DL-UCM-ST).

In order to appreciate the variability of learned image features, the final DL-UCM-ST model was used to generate and visualize post-convolution layer activations for all image tiles. Each image tile was presented to the DL-UCM-ST model as input, and activation values for each of the 2048 nodes in the final post-convolution layer were calculated. Each node in this layer is activated by a learned image feature, and in this way, the activations vector represents a complex summary of detected image features. Once calculated, activation vectors across all tiles were then mapped with the dimensionality reduction technique UMAP [32].

### Evaluating deep learning model performance on TCGA cohort

Digital slides from the thyroid cancer cohort of TCGA (THCA) were downloaded using the official TCGA data portal, restricted to slides with an annotated tumor subtype of “Papillary adenocarcinoma, NOS” (PTC-NOS), “Papillary adenocarcinoma, columnar cell” (PTC-tall) or “Papillary carcinoma, follicular variant” (PTC-FV). Two slides were excluded due to poor slide quality. The final dataset comprised 354 PTC-NOS, 38 PTC-tall, and 105 PTC-FV (Fig. 1a). Regions of interest were annotated by pathologists and tiles were extracted and processed as above. The deep learning model trained on our institutional dataset was then used to create subtype predictions for all slides in the TCGA cohort. Since TCGA samples do not contain benign tissue, model predictions were constrained to only PTC-classic, PTC-EFG, or NIFTP. Predictions generated by this model were compared between annotated diagnosis using Chi-squared tests. BRS for TCGA slides were downloaded from data published by Agrawal [1]. BRS was available for 386 tumors (269 BRAF^V600E^-like, 117 RAS-like). BRS was compared between categories using both Chi-squared tests (when scores were dichotomized to either BRAF^V600E^-like or RAS-like) and ANOVA (when comparing linear score values). Post-convolution layer activations were calculated for all TCGA slides and visualized using UMAP.

### Training a deep learning model on the TCGA cohort to predict BRS

We then trained a deep learning model on the TCGA cohort using BRS as a linear outcome. As previously, the TCGA dataset was split into thirds for the purpose of performing cross-validation. The model was built using the same parameters as previously determined, except with mean squared error as the loss function, and the number of epochs was redetermined in the first *k-*fold; the optimal number of epochs was determined to be two, which was then used for the other two *k*-folds. Predicted BRS in the validation cohorts were calculated and aggregated across *k*-folds, with performance assessed by calculating *R*^2^. BRS was then dichotomized to either RAS-like (positive score) or BRAF^V600E^-like (negative score), and model performance was assessed on these dichotomized outcomes using receiver operator curves and calculating AUCs. A final model was then trained across the entire TCGA dataset (DL-TCGA-BRS), which was then used to generate BRS predictions on our institutional dataset. Slide-level BRS predictions were generated by averaging all tile-level predictions for a given slide. We compared predicted BRS among the FAs, NIFTP, PTC-EFG, and PTC-classic subtypes using both Chi-squared and ANOVA as described. We again calculated post-convolution layer activations using the trained BRS model, which were subsequently mapped using UMAP.

### Mosaic maps

In order to better appreciate image features detected by the deep learning models, mosaic maps were created from post-convolution layer activations. Activations were calculated for each tile as previously described and mapped with UMAP. These activations were overlaid onto a 50 × 50 grid, and for each grid space containing mapped tiles, a tile image was chosen for display. When multiple tiles were eligible for display in a given grid space, the tile nearest to centroid with respect to post-convolution layer activations was displayed.

## Results

### Deep learning models distinguish tumor subtypes

A deep learning model was trained on 115 pathologist-annotated slides in a local institutional dataset, with distribution of diagnoses as reported in Table 1, to predict tumor subtype (DL-UCM-ST) (Fig. 1c, d). Aggregated cross-validated performance revealed high sensitivity and specificity for NIFTP (sensitivity 89.4%, specificity 89.7%), high sensitivity for PTC-classic (sensitivity 94.7%, specificity 79.2%), and high specificity but low sensitivity for both PTC-EFG (sensitivity 39.1%, specificity 97.8%) and benign FA (sensitivity 42.9%, specificity 100%).

**Fig. 2:**
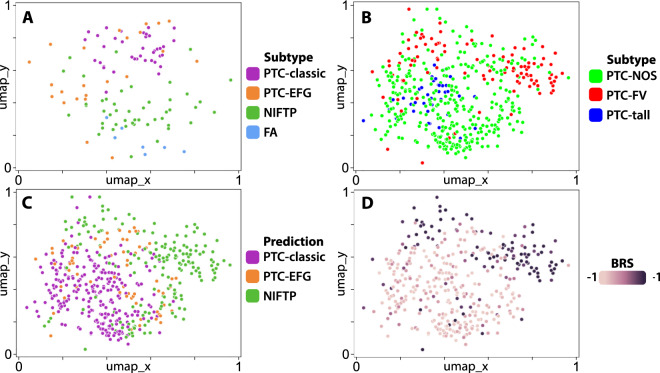
UMAPs of post-convolution activations generated by deep learning models trained to predict tumor subtype. **a** An Xception-based deep learning model was trained on 115 slides from an institutional database of thyroid cancer to predict tumor subtype (DL-UCM-ST). Post-convolution layer activations were calculated on all tiles and mapped with UMAP. For each slide, the tile nearest to centroid was plotted and labeled according to its tumor subtype. NIFTP slides can be seen to cluster distinctly from the PTC subtypes, clustering near follicular adenomas. Several PTC-EFGs cluster near PTC-classics, but approximately half are found between the NIFTP and PTC-classic clusters. **b** The DL-UCM-ST model was used to generate slide-level predictions in the TCGA thyroid cohort. Slides with follicular-pattern cluster together, but demonstrate overlap with slides labeled as “Papillary thyroid carcinoma, not otherwise specified” (PTC-NOS). **c** Same as (**b**), with slides labeled according to their predicted subclass. Many of the true PTC-EFG tumors are predicted to be NIFTPs, as can be seen by comparing this plot to (**b**). Tumors which are predicted to be NIFTP cluster distinctly from those predicted to be PTC-classic. Many of the tumors falling on the border of these two clusters are predicted to be PTC-EFGs. **d** Same as (**b**), with slides labeled according to their BRS, as calculated by Agarwal using a 71-gene mRNA expression signature [1]. Tumors with BRS > 0 are RAS-like, and BRS < 0 indicate BRAF^V600E^-like phenotype. Tumors with high BRS, and thus RAS-like phenotype, are highly associated with the cluster of tumors predicted to be NIFTPs, as can be appreciated by comparing this plot with *C*. The majority of BRAF^V600E^-like tumors are predicted to be either PTC-classics or PTC-EFGs.

In order to better understand the relationship of detected histologic features between tumor subtypes, a UMAP plot was generated from post-convolution layer activations for each tile across all slides (Fig. 2a). For each slide, the tile nearest to centroid was identified, displayed on the plot, and labeled according to its subtype. NIFTPs clustered distinctly from PTCs, with benign follicular adenomas demonstrating similarity to NIFTPs in their distribution. PTC-classics clustered distinctly from NIFTPs, whereas PTC-EFGs demonstrated greater spread; approximately half of PTC-EFGs possessed an activation signature similar to PTC-classics, while the other half were closer to, yet still distinct from, NIFTPs.

### Assessment of tumor subtype prediction with TCGA

To assess the ability of this model to differentiate thyroid subclasses on an external validation set, predictions were generated for all slides in the TCGA thyroid cancer cohort (THCA) using the final trained DL-UCM-ST model, with predictions constrained to only PTC-classic, PTC-EFG, and NIFTP (Table 2). Interestingly, most of the follicular-patterned neoplasms were predicted to be NIFTP rather than PTC-EFG (71.7% vs 17.2%). Examining mutation status revealed a strong association between NIFTP prediction and *RAS* aberrations, with 86.0% of *RAS*-mutant tumors predicted to be NIFTP, compared to 30.9% NIFTP prediction within *RAS* non-mutants (*P* < 0.0001). Similarly, the occurrence of PTC-classic subtype prediction within *RAS*-mutant tumors was lower than *RAS* non-mutants (4.0% vs. 51.3%, *P* < 0.0001).

**Table 2:** Subtype predictions across TCGA subtype labels, by BRAF/RAS mutation status. Table excludes a single dual RAS/BRAF mutant PTC-NOS.

	Molecular alteration	Predictions		
		PTC-classic	PTC-EFG	NIFTP
PTC-FV	*RAS* mutant	0 (0%)	3 (10%)	27 (90%)
	*BRAF* mutant	8 (42%)	6 (32%)	5 (26%)
	*RAS*/*BRAF* non-mutant	3 (6%)	8 (16%)	39 (78%)
PTC-NOS	*RAS* mutant	2 (10%)	2 (10%)	16 (80%)
	*BRAF* mutant	145 (63%)	33 (14%)	54 (23%)
	*RAS*/*BRAF* non-mutant	24 (30%)	25 (31%)	31 (39%)
PTC-tall	*RAS* mutant	0 (0%)	0 (0%)	0 (0%)
	*BRAF* mutant	30 (94%)	2 (6%)	0 (0%)
	*RAS*/*BRAF* non-mutant	4 (100%)	0 (0%)	0 (0%)
PTC-FV	BRAF-like	9 (47%)	8 (42%)	2 (11%)
	RAS-like	1 (1%)	6 (9%)	61 (90%)
PTC-NOS	BRAF-like	125 (57%)	41 (19%)	55 (25%)
	RAS-like	6 (13%)	7 (15%)	35 (73%)
PTC-tall	BRAF-like	27 (93%)	2 (7%)	0 (0%)
	RAS-like	1 (100%)	0 (0%)	0 (0%)

An additional UMAP was generated as previously, using post-convolution layer activations calculated from the subtype prediction model (Fig. 2b). PTC-EFGs cluster together with a moderate amount of overlap with PTC-NOS. PTC-tall tumors cluster separately from PTC-EFGs within the larger PTC-NOS cluster. The UMAP was then labeled with slide-level predictions (Fig. 2c). Slides predicted to be PTC-classic clustered distinctly from those predicted to be NIFTP, with a small degree of overlap. Slides predicted to be PTC-EFG lie predominantly at the border between predicted-NIFTP and predicted-PTC-classic.

Next, given the association between subtype prediction and *RAS* mutation status, we investigated the role of the BRAF-RAS axis on detectable histologic features by comparing BRS among predicted subtypes (Fig. 2d). A positive BRS (RAS-like) score was found to be strongly associated with NIFTP prediction. Within follicular-patterned PTCs, tumors with positive BRS were 8.5 times as likely to carry an NIFTP prediction than tumors with negative BRS (89.7% vs 10.5%, *P* < 0.0001). Tumors predicted to be PTC-EFG were more likely to contain BRAF^V600E^-like signatures than RAS-like signatures (79.7% vs 20.3%, *P* < 0.0001), as were predicted PTC-classics (95.3% vs 4.7%, *P* < 0.0001).

### BRAF-RAS score prediction and association with tumor subtype

**Fig. 3:**
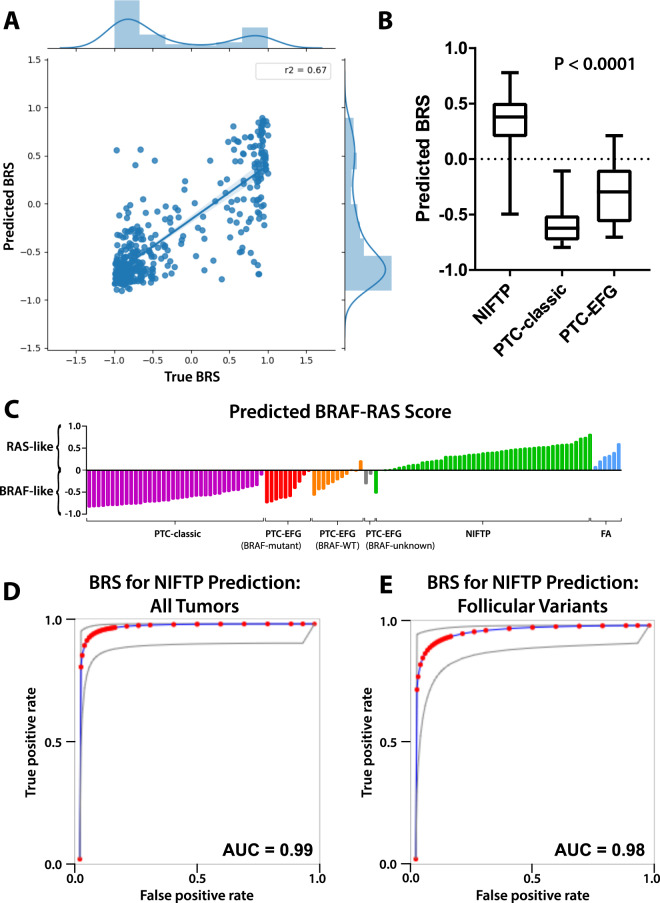
Performance of deep learning models trained to predict BRS. **a** Xception-based deep learning models were trained on the TCGA thyroid cohort to predict BRS as a linear outcome using threefold cross validation. For each slide in the left-out validation sets, slide-level score predictions were generated by averaging predictions across constituent tiles. Predicted BRS was plotted against true BRS for each *k*-fold, with *R*^2^ calculated 0.67, 0.73, and 0.61 for each of the *k-*folds. The three plots were then aggregated across *k*-folds as shown in (**a**), with an aggregated *R*^2^ of 0.67. **b** A final model was generated by training across the entire TCGA thyroid cohort (DL-TCGA-BRS) and was used to predict BRS for slides in the institutional dataset. Distribution of BRS predictions can be seen across NIFTP, PTC-classic, and PTC-EFG slides. NIFTP tumors had significantly higher predicted BRS, indicating a more RAS-like phenotype, than either of the PTC subclasses, as calculated using ANOVA (*P* < 0.0001). **c** BRS predictions across slides in the institutional dataset, separated by tumor subtype. NIFTPs are near universally predicted to be RAS-like, with PTC-classics predominantly BRAF^V600E^-like. PTC-EFG tumors are largely BRAF^V600E^-like but demonstrate somewhat greater heterogeneity. **d** Predicted BRS is an excellent discriminator of NIFTP status; using predicted BRS as a surrogate for NIFTP prediction, the test has an AUC of 0.99 when applied across all PTC-classic, PTC-EFG, and NIFTP tumors in the institutional dataset. **e** Used as a discriminator solely between PTC-EFG and NIFTP tumors, predicted BRS has an AUC of 0.98.

In a reverse strategy, we then trained a deep learning model on the TCGA cohort to predict BRS as a linear outcome, with the goal of generating predicted scores on our internal dataset (Fig. 1e, f). R^2^ of slide-level predictions across the three *k*-folds were 0.67, 0.73, and 0.61, with a scatterplot of performance aggregated across *k*-folds shown in Fig. 3a. Dichotomizing BRS predictions to either BRAF^V600E^-like or RAS-like resulted in aggregated AUC of 0.935 across the three *k*-folds. While we were unable to validate the BRS directly on our institutional dataset as the BRS weights are unpublished, we generated BRS predictions and compared these predictions to a subset of tumors with known *BRAF*^V600E^ and/or *RAS* mutation status. All 34 *BRAF*^V600E^ mutant tumors had a BRS of less than 0, indicating a predicted BRAF^V600E^-like phenotype. Similarly, all 21 *RAS*-mutant tumors had a BRS of greater than 0, indicating a predicted RAS-like phenotype.

Predicted BRS was highly associated with both FA and NIFTP, to a higher degree even than the prior model trained to predict tumor subtype directly (Fig. 3b, c). NIFTPs had a mean predicted BRS of 0.35 ± 0.03, compared to a predicted score of −0.60 ± 0.03 in PTC-classics and −0.30 ± 0.06 in PTC-EFGs. Only one NIFTP was predicted to have BRAF^V600E^-like, rather than RAS-like, phenotype (BRS −0.50). *BRAF*^V600E^ mutant PTC-EFGs were all predicted to have negative BRS (mean −0.49), while *BRAF* nonmutant PTC-EFGs were more variable (mean −0.17), though still distinguishable from NIFTP (mean 0.35; *P* < 0.0001). As a discriminator of NIFTP vs. non-NIFTP across 107 PTC-classics, PTC-EFGs, and NIFTPs, the test has an AUC of 0.99 and a sensitivity and specificity of 97.9% and 96.6% (Fig. 3d). As a test to identify NIFTP within only follicular-patterned neoplasms, the BRS prediction model has an AUC of 0.98, with a sensitivity and specificity of 97.9% and 90.0% (Fig. 3e).

### Histologic features of the BRAF-RAS spectrum

**Fig. 4:**
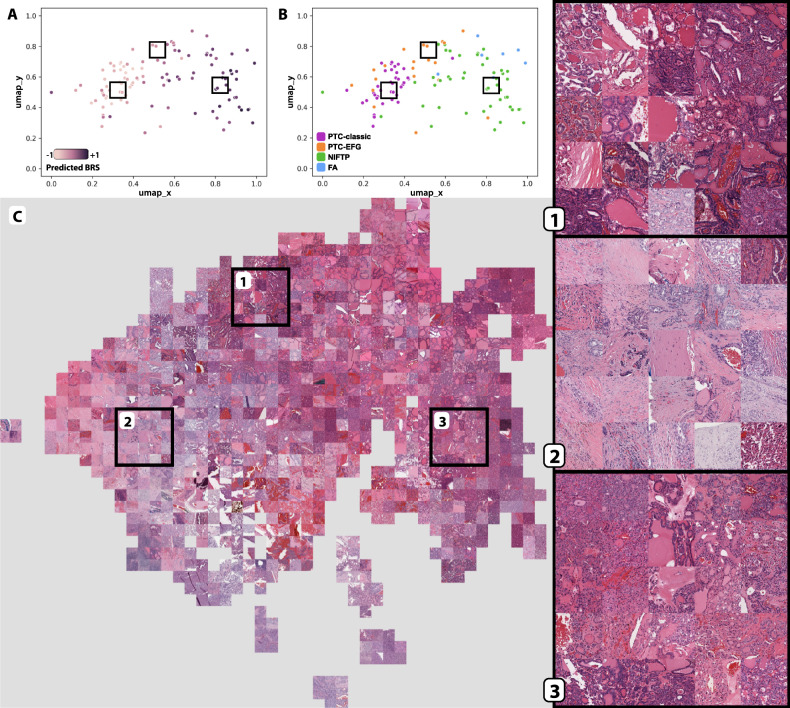
Mosaic map of institutional cohort, mapped with post-convolution activations generated from the DL-TCGA-BRS model. **a** Post-convolution layer activations for all image tiles in the institutional cohort were calculated by using a model trained on TCGA slides to predict BRS. Tile activations were then mapped using UMAP, and centroid tiles for each slide were plotted as with Fig. 2. Slides are colored according to predicted BRS, with dark purple indicating high predicted BRS and light purple indicating low predicted BRS. Boxes correspond to the areas of interest highlighted in (**c**). **b** Same as (**a**), with slides labeled according to tumor subtype. **c** All tiles, rather than just slide-level centroid tiles, were plotted using UMAP as with (**a** and **b**). A mosaic map was then created from this UMAP plot by replacing plotted tiles with their corresponding images, organized into a grid pattern. Three areas were chosen for magnified display.

In an effort to describe the histologic features detected by the BRS prediction model, final layer activations were generated from this model across slides in the institutional cohort and plotted using UMAP (Fig. 4). Predicted BRS neatly increases near-linearly from left to right, with the highest predicted BRS occurring within NIFTP clusters. Overall, the tumor subtypes clustered similarly to the model trained directly to tumor subtype (Fig. 2), with NIFTPs clustering separately from PTCs, follicular adenomas clustering near/within NIFTPs, and PTC-EFGs falling largely between PTC-classics and NIFTPs, with some PTC-EFGs clustering within PTC-classics. A mosaic map was generated from these predictions, with three areas corresponding to high PTC-EFG, PTC-classic, and NIFTP density magnified to help illustrate some of the histologic features observed in these two categories (Fig. 4c). Tiles within area 1, predominantly from PTC-EFGs, are marked by variably-sized follicles with some stromal fibrosis, dark-stained colloid with occasional scalloping, and irregular, wrinkled, cleared nuclei. Area 2 contains mostly PTC-classic tiles and displays extensive stromal fibrosis with infiltration by neoplastic follicles and papillae. Area 3 is enriched for tiles from NIFTP tumors with high predicted BRS, showing predominantly tightly packed microfollicles with variable amounts of nuclear atypia.

**Fig. 5:**
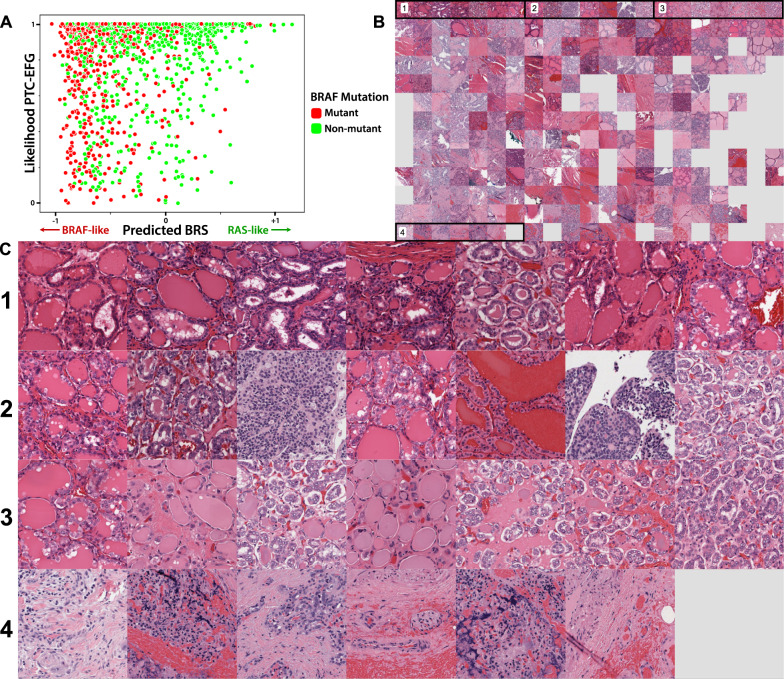
Map of predicted BRAF-RAS score and subtype prediction within PTC-EFG tumors. **a** Tiles from PTC-EFG slides were mapped in two dimensions, according to predicted BRS on the *x*-axis (−1 on the left to +1 on the right) and PTC-EFG subtype prediction on the *y*-axis (low likelihood of PTC-EFG subtype at the bottom, high likelihood at the top). Tiles are annotated according to *BRAF* mutation status of their corresponding slides and were subsampled to only include 2000 points for display. **b** Tile points as calculated in *A* were replaced with corresponding tile images, organized into a grid. Four areas are selected for magnified display in (**c**). **c** Magnified display of four areas noted in (**b**). Areas 1, 2, and 3 all correspond to tiles with high-likelihood of PTC-EFG prediction. Area 1 contains tiles with low predicted BRS (BRAF^V600E^-like), with tiles increasing in predicted BRS from left to right. In this way, these are tiles with image features specific to the PTC-EFG subtype. Area 2 contains tiles with intermediate predicted BRS, increasing left to right, indicating tiles sufficient for identification of a follicular pattern but nonspecific with regard to BRAF-RAS features. Area 3 contains tiles with high predicted BRS (RAS-like), increasing left to right, indicating tiles that are more consistent with a RAS-like phenotype and NIFTP diagnosis.

Finally, to explore the histologic landscape of the PTC-EFG subtype in greater detail, both BRS and tumor subtype were predicted using the models mentioned above for all PTC-EFG tiles in the institutional cohort (Fig. 5). Tiles were plotted according to predicted BRS on the *x*-axis and likelihood of PTC-EFG prediction on the *y*-axis. By examining the top of this graph, one can visualize how the histology of PTC-EFG tumors is predicted to vary according to BRS status. Tiles in the top-left corner (area 1), corresponding to PTC-EFG subtype prediction and a BRAF^V600E^-like phenotype, possess variably-sized follicles and occasional scalloped colloid. Tiles in the top-right corner (area 3), still predicted to be PTC-EFG but with a more RAS-like phenotype, display tightly packed microfollicles with more pronounced nuclear clearing. The bottom-left corner (area 4) contains tiles with low likelihood of PTC-EFG prediction and a more PTC-classic appearance exemplified by collagenous stroma with infiltrative neoplastic cells.

## Discussion

In order to test our hypothesis that NIFTPs are associated with RAS-like BRS, we began with evaluating slides of thyroid neoplasms in the THCA cohort of TCGA. Our aim was to assess whether NIFTPs had higher BRS (RAS-like) on average than PTCs. Since TCGA annotations do not account for the NIFTP subtype, we created a deep learning model, trained on an institutional dataset to predict thyroid neoplasm subtype, that could generate modern diagnostic predictions for slides in TCGA. Our goal was to assess whether BRS among tumors predicted to be NIFTP were significantly different from BRS among tumors predicted to be PTCs.

Our training set was composed of 115 slides across 4 different subtypes—much lower than the 497 slides in TCGA. For this reason, we anticipated that this first deep learning model would be unlikely to generate perfectly generalizable predictions, and in fact, we did not possess an external validation dataset to assess its predictive accuracy. Rather, we used this model as a first-pass attempt to determine if an association between NIFTPs and BRS existed by assessing whether tumors with higher BRS (RAS-like expression) were more likely to be predicted NIFTPs. Within follicular-patterned tumors, RAS-like tumors were more than eight times as likely to be predicted as NIFTP by this deep learning model. Tumors predicted to be PTC-EFG were approximately 3.9 times more likely to contain BRAF^V600E^-like signatures than RAS-like signatures, and predicted PTC-classics were more than 20 times as likely to contain BRAF^V600E^-like signature. Taken together, this first deep learning model demonstrated that histologic features of NIFTP are more likely to be seen in tumors with RAS-like gene expression than BRAF^V600E^-like expression.

To further test our hypothesis that NIFTPs are defined by RAS*-*like gene expression, we then sought to determine whether a model trained to directly predict BRS—irrespective of tumor subtype—would be sufficient to differentiate NIFTPs from other thyroid neoplasms. We started by testing the feasibility of training a model to predict BRS by performing cross-validation training on TCGA, and found that histologic features associated with BRS were successfully detected with deep learning, with aggregated predictive accuracy for these cross-validated models demonstrating an *R*^2^ of 0.67. As a dichotomized variable, the cross-validated performance within the TCGA cohort was able to distinguish RAS-like expression from BRAF^V600E^-like expression with an AUC of 0.94.

After determining that BRS could be predicted from tumor histology, we trained a final model across the entire TCGA dataset and generated BRS predictions for our internal cohort. While we were unable to validate the scores directly, as the components and weights of the gene expression score are not published, we found that predicted RAS-like gene expression was highly specific for the NIFTP subtype within malignant neoplasms. Using predicted RAS-like gene expression as a sole discriminator of NIFTP status, the test performed exceedingly well with an AUC of 0.99 across all malignant slides in our dataset, and 0.98 when restricted to follicular-patterned malignant neoplasms. In fact, only one NIFTP had a grossly BRAF^V600E^-like prediction. Interestingly however, this particular NIFTP was atypical in that it was an oncocytic neoplasm, for which the NIFTP diagnosis has not been adequately assessed. In addition, review of the slide demonstrated the presence of focal papillary architecture, which under the newest diagnostic criteria should preclude the diagnosis of NIFTP [15]. Last, next generation sequencing of this neoplasm was negative for both *BRAF*^V600E^ and *RAS* mutations. Therefore, it is not entirely certain why this tumor resulted in a BRAF^V600E^-like prediction in this study.

We found remarkable similarity between NIFTPs and benign FAs, both with respect to overall histologic features as well as predicted BRS. When examining post-convolution activations generated by DL-UCM-ST, we observed significant overlap in the image features detected in NIFTPs and FAs, as evidenced by the UMAP shown in Fig. 2a. NIFTPs and FAs also clustered together when post-convolution activations generated by DL-TCGA-BRS were plotted with UMAP (Fig. 4b). Furthermore, as with NIFTPs, the DL-TCGA-BRS model predicted that all seven FAs had RAS-like BRS. These findings suggest that the histologic features seen in NIFTP have more in common with benign FAs than malignant PTCs, which is consistent with the observation that these indolent neoplasms have benign behavior.

One of the great challenges of successful deep learning models is understanding the image features that have been learned after training. In order to assess the histologic differences associated with RAS-like and BRAF^V600E^-like gene expression signatures, we generated mosaic maps using post-convolution layer activations from our deep learning model and visually inspected image tiles associated with these two classes. On the whole, image tiles highly predictive of BRAF^V600E^-like morphology in PTC-EFGs tended to have variably-sized and/or dilated follicles, often associated with stromal fibrosis, and prominent nuclear membrane irregularities and/or clearing. Image tiles highly predictive of RAS-like gene expression in NIFTPs tended to have more tightly packed microfollicles with variable amounts of nuclear atypia. While NIFTPs are typically thought of as having nuclear features and follicles on the same spectrum as PTC, these results suggest that the follicles and high-power cellular features are distinct between NIFTP and PTC-EFG, particularly in the presence of a *BRAF*^V600E^ mutation.

Taken together, these results demonstrate that the histologic features associated with BRAF-RAS spectrum are detectable by deep learning, and furthermore, that automated detection of these image features can aid in distinguishing indolent NIFTP from PTCs. The fact that a deep learning model trained to predict BRS, irrespective of subtype, was able to successfully distinguish NIFTPs from other follicular thyroid neoplasms suggests that the gene expression score is reflective of underlying tumor biology, and the image features associated with these biological processes are sufficiently prevalent to allow for differentiation of these subtypes.

Identifying a neoplasm as either RAS-like or BRAF^V600E^-like may aid in distinguishing indolent neoplasms from their more aggressive counterparts, providing better prognostication and safer de-escalation of therapy. This is well exemplified by the recent report of a thyroid neoplasm, initially diagnosed as NIFTP, which unexpectedly metastasized and was found to possess a *BRAF*^V600E^ mutation [20]. Current NIFTP criteria exclude tumors harboring *BRAF*^V600E^ or other aggressive (*TERT* promoter or *TP53*) mutation. It is possible that routine incorporation of molecular diagnostics, which may include both mutational testing and either direct gene expression assessment or estimation of BRS through deep learning, may assist with ensuring that the NIFTP diagnosis is restricted to indolent neoplasms. Given the strong association between predicted RAS-like phenotype and both NIFTPs and benign FAs, these results advocate for further validation of the use of BRS testing as an aid for diagnosing follicular-patterned neoplasms, either directly or through a deep learning-based prediction surrogate. With further validation, we propose that a BRAF^V600E^-like signature should exclude the diagnosis of NIFTP. This suggestion is consistent with a recent proposal by Johnson to broadly reclassify follicular-patterned neoplasms into RAS-like (NIFTP, FA, IE-PTC-FV) and BRAF^V600E^-like (PTC-EFG, classic PTC) [5].

Of note, the deep learning approach described herein is limited by a lack of evaluation for circumscription or encapsulation, an assessment of which is necessary before a diagnosis of NIFTP can be rendered in order to avoid missing IE-PTC-FVs. This is due both to the nature of the deep learning method—which analyzes tiles at a fixed ×10 magnification—and because the pathologist annotations on the physical slide media preclude accurate assessment of tumor capsules. For this reason, we do not envision that a deep learning tool such as what we have described would be sufficient to render a diagnosis alone. Rather, we envision assessment of BRS status as an ancillary tool for pathologists at the time of diagnosis.

In summary, the histologic features associated with the BRAF-RAS gene expression spectrum are detectable by deep learning and can aid in distinguishing indolent NIFTP from PTCs. Given the high degree of interobserver variability in the diagnosis of the follicular-patterned thyroid neoplasms resulting in the need for simplified definitions and numerous exclusion criteria, the prospect of using a deep learning model to help standardize the distinction between RAS-like and BRAF^V600E^-like histologic and nuclear features, or at least aid in the diagnosis of particularly difficult cases, is appealing. These results advocate for further validation of the use of BRS testing as a diagnostic aid for follicular-patterned neoplasms, either calculated directly or performed through a deep-learning based prediction surrogate. Improving identification of NIFTP by assessment of the BRAF-RAS axis may help ensure it remains an indolent neoplasm, assist with safe de-escalation of therapy, and prevent overtreatment.

## Supplementary information

Supplementary Information

## References

[1] Cancer Genome Atlas Research Network. (2014). Integrated genomic characterization of papillary thyroid carcinoma. Cell.

[2] Daniels GH (2011). What if many follicular variant papillary thyroid carcinomas are not malignant? A review of follicular variant papillary thyroid carcinoma and a proposal for a new classification. Endocr Pract.

[3] Omur O, Baran Y (2014). An update on molecular biology of thyroid cancers. Crit Rev Oncol Hematol.

[4] Seethala RR, Baloch ZW, Barletta JA, Khanafshar E, Mete O, Sadow PM (2018). Noninvasive follicular thyroid neoplasm with papillary-like nuclear features: a review for pathologists. Mod Pathol.

[5] Johnson DN, Furtado LV, Long BC, Zhen CJ, Wurst M, Mujacic I (2018). Noninvasive follicular thyroid neoplasms with papillary-like nuclear features are genetically and biologically similar to adenomatous nodules and distinct from papillary thyroid carcinomas with extensive follicular growth. Arch Pathol Lab Med.

[6] Rivera M, Ricarte-Filho J, Knauf J, Shaha A, Tuttle M, Fagin JA (2010). Molecular genotyping of papillary thyroid carcinoma follicular variant according to its histological subtypes (encapsulated vs infiltrative) reveals distinct BRAF and RAS mutation patterns. Mod Pathol.

[7] Howitt BE, Jia Y, Sholl LM, Barletta JA (2013). Molecular alterations in partially-encapsulated or well-circumscribed follicular variant of papillary thyroid carcinoma. Thyroid..

[8] Gupta S, Ajise O, Dultz L, Wang B, Nonaka D, Ogilvie J (2012). Follicular variant of papillary thyroid cancer: encapsulated, nonencapsulated, and diffuse: distinct biologic and clinical entities. Arch Otolaryngol Head Neck Surg.

[9] Ghossein R (2010). Encapsulated malignant follicular cell-derived thyroid tumors. Endocr Pathol.

[10] Wreesmann VB, Ghossein RA, Hezel M, Banerjee D, Shaha AR, Tuttle RM (2004). Follicular variant of papillary thyroid carcinoma: genome-wide appraisal of a controversial entity. Genes Chromosomes Cancer.

[11] Xu B, Ghossein R (2015). Encapsulated thyroid carcinoma of follicular cell origin. Endocr Pathol.

[12] Guney G, Tezel GG, Kosemehmetoglu K, Yilmaz E, Balci S, Ersoy R (2014). Molecular features of follicular variant papillary carcinoma of thyroid: comparison of areas with or without classical nuclear features. Endocr Pathol.

[13] Vivero M, Kraft S, Barletta JA (2013). Risk stratification of follicular variant of papillary thyroid carcinoma. Thyroid..

[14] Nikiforov YE, Seethala RR, Tallini G, Baloch ZW, Basolo F, Thompson LDR (2016). Nomenclature revision for encapsulated follicular variant of papillary thyroid carcinoma: a paradigm shift to reduce overtreatment of indolent tumors. JAMA Oncol.

[15] Nikiforov YE, Baloch ZW, Hodak SP, Giordano TJ, Lloyd RV, Seethala RR (2018). Change in diagnostic criteria for noninvasive follicular thyroid neoplasm with papillarylike nuclear features. JAMA Oncol.

[16] Ferris RL, Baloch Z, Bernet V, Chen A, Fahey TJ, Ganly I (2015). American thyroid association statement on surgical application of molecular profiling for thyroid nodules: current impact on perioperative decision making. Thyroid.

[17] Elsheikh TM, Asa SL, Chan JK, DeLellis RA, Heffess CS, LiVolsi VA (2008). Interobserver and intraobserver variation among experts in the diagnosis of thyroid follicular lesions with borderline nuclear features of papillary carcinoma. Am J Clin Pathol.

[18] Lloyd RV, Erickson LA, Casey MB, Lam KY, Lohse CM, Asa SL (2004). Observer variation in the diagnosis of follicular variant of papillary thyroid carcinoma. Am J Surg Pathol.

[19] Hirokawa M, Carney JA, Goellner JR, DeLellis RA, Heffess CS, Katoh R (2002). Observer variation of encapsulated follicular lesions of the thyroid gland. Am J Surg Pathol.

[20] Cho U, Mete O, Kim M-H, Bae JS, Jung CK (2017). Molecular correlates and rate of lymph node metastasis of non-invasive follicular thyroid neoplasm with papillary-like nuclear features and invasive follicular variant papillary thyroid carcinoma: the impact of rigid criteria to distinguish non-invasive follicular thyroid neoplasm with papillary-like nuclear features. Mod Pathol.

[21] Xu B, Farhat N, Barletta JA, Hung YP, Biase D, Casadei GP (2018). Should subcentimeter non-invasive encapsulated, follicular variant of papillary thyroid carcinoma be included in the noninvasive follicular thyroid neoplasm with papillary-like nuclear features category?. Endocrine..

[22] Xu B, Reznik E, Tuttle RM, Knauf J, Fagin JA, Katabi N (2019). Outcome and molecular characteristics of non-invasive encapsulated follicular variant of papillary thyroid carcinoma with oncocytic features. Endocrine..

[23] Xu B, Tallini G, Scognamiglio T, Roman BR, Tuttle RM, Ghossein RA (2017). Outcome of large noninvasive follicular thyroid neoplasm with papillary-like nuclear features. Thyroid.

[24] Kather JN, Heij LR, Grabsch HI, Loeffler C, Echle A, Muti HS (2020). Pan-cancer image-based detection of clinically actionable genetic alterations. Nature Cancer..

[25] Coudray N, Ocampo PS, Sakellaropoulos T, Narula N, Snuderl M, Fenyö D (2018). Classification and mutation prediction from non–small cell lung cancer histopathology images using deep learning. Nat Med.

[26] Sha L, Osinski B, Ho I, Tan T, Willis C, Weiss H (2019). Multi-field-of-view deep learning model predicts nonsmall cell lung cancer programmed death-ligand 1 status from whole-slide hematoxylin and eosin images. J Pathol Inform.

[27] Zhang H, Ren F, Wang Z, Rao X, Li L, Hao J, et al., editors. Predicting Tumor Mutational Burden from Liver Cancer Pathological Images Using Convolutional Neural Network. 2019 IEEE International Conference on Bioinformatics and Biomedicine (BIBM); 2019 18-21 Nov. 2019.

[28] Tsou P, Wu CJ (2019). Mapping driver mutations to histopathological subtypes in papillary thyroid carcinoma: applying a deep convolutional neural network. J Clin Med..

[29] Fu Y, Jung AW, Torne RV, Gonzalez S, Vöhringer H, Shmatko A (2020). Pan-cancer computational histopathology reveals mutations, tumor composition and prognosis. Nat Cancer.

[30] Martín A, Agarwal A, Barham P, Brevdo E, Chen Z, Citro C, et al. TensorFlow: Large-scale machine learning on heterogeneous systems. 2015; https://www.tensorflow.org/about/bib.

[31] Chollet F, editor Xception: Deep Learning with Depthwise Separable Convolutions. 2017 IEEE Conference on Computer Vision and Pattern Recognition (CVPR); 2017 21-26 July 2017.

[32] McInnes L, Healy J, Melville J. UMAP: Uniform manifold approximation and projection for dimension reduction. 2018. arXiv:1802.03426 [stat.ML].

