# Supplementary material for: Deep learning prediction of BRAF-RAS gene expression signature identifies noninvasive follicular thyroid neoplasms with papillary-like nuclear features
Source: Mod Pathol. 2020 Dec 10;34(5):862–74. doi: 10.1038/s41379-020-00724-3 (PMC8064913; doi:10.1038/s41379-020-00724-3)
Supplement: Supplementary file 1 — Supplementary Information [file 41379_2020_724_MOESM1_ESM.docx]

**Supplementary Information**

| Hyperparameter | Value |
| --- | --- |
| Learning rate | 0.0001 |
| Batch size | 16 |
| Pooling | “max” |
| Hidden layers | 0 |
| Epochs | 5^*^, 2^†^ |

^*^Used for DL-UCM-ST model

^†^Used for DL-TCGA-BRS model

**Supplementary Table S1**. *Hyperparameters used for deep learning models.*
